# Clinical and Microbiological Analysis of Hospital-Acquired Pneumonia Among Patients With Ischemic Stroke: A Retrospective Outlook

**DOI:** 10.7759/cureus.15214

**Published:** 2021-05-24

**Authors:** Muhammad Adnan Wattoo, Muhammad Tabassum, Kiran R Bhutta, Zainab Rafi, Mehwish Kaneez, Mustafa Tauseef Razzaq, Rafay Rizwan, Zoya Sarwar, Muhammad Usama Sajid, Fatima Rafique Bhutta

**Affiliations:** 1 Neurology, Sialkot Medical College, Sialkot, PAK; 2 Internal Medicine, Islam Medical and Dental College, Sialkot, PAK; 3 Internal Medicine, Rawalpindi Medical University, Rawalpindi, PAK; 4 Internal Medicine, Rashid Latif Medical College, Lahore, PAK

**Keywords:** hospital-acquired pneumonia, ischemic stroke, antimicrobial resistance, microbiological analysis, clinical parameters

## Abstract

Background

Hospital-acquired pneumonia (HAP) is a leading cause of morbidity and mortality in patients with ischemic stroke. Our study aims to explore the clinical and microbiological aspects (culture and sensitivity) of stroke patients with a confirmed diagnosis of HAP.

Methodology

This retrospective cross-sectional study included a total of 232 patients with a confirmed diagnosis of HAP following ischemic stroke. HAP was diagnosed based on the timing of onset of symptoms and chest X-ray. Patients were evaluated for their demographic details and a myriad of clinical parameters including dysphagia, mechanical ventilation, mortality, spontaneous pneumothorax, and Glasgow coma scale (GCS) score. The frequencies of different microorganisms isolated from the tracheal secretions were reported. Thereafter, the percentages of resistant isolates against a plethora of antimicrobial agents were tabulated.

Results

Out of 232 patients, 110 were males and 122 were females with a mean age of 58.79 ± 8.62 years. Dysphagia and mechanical ventilation were present in 66.4% and 72%, respectively. The mortality rate was 30.6%. *Klebsiella pneumoniae* was the most frequently isolated organism (28.9%), followed by *Escherichia coli* (24.5%), and *Pseudomonas aeruginosa* (23.3%). The antimicrobial resistance patterns of most of the isolates against different antibiotics were alarmingly high.

Conclusions

Dysphagia and mechanical ventilation are frequently present in patients of ischemic stroke with associated HAP. The antimicrobial resistance patterns of the isolated organisms are a cause of major concern. This necessitates the need for proper sanitation and the careful use of antibiotics.

## Introduction

Ischemic stroke represents a detrimental cerebrovascular disorder characterized by acute compromise of blood flow [1]. Clot formation in the form of thrombus and clot migration via embolization of a thrombus causes the rapid development of focal neurological deficits [2]. Around the globe, stroke affects 13.7 patients while 5.5 million individuals succumb to its complications [3,4]. Nearly 50% of the survivors become handicapped for a lifetime [4]. Furthermore, multiple comorbidities including hypertension, diabetes, and cardiovascular disorders increase the likelihood of developing various complications [5]. Hospital-acquired pneumonia (HAP), dysphagia, cardiovascular complications, hemiplegia, and urinary incontinence are some of the early complications that occur after a stroke attack [6]. In contrast, venous thromboembolism, bedsores, urinary tract infections, gastrointestinal complications, and psychiatric are the late complications [4,6].

Although neurological complications are the foremost cause of mortality among stroke-affected patients, HAP has been reported to be a common and serious complication [7]. After seven days of a stroke attack, HAP is the most common complication affecting more than 25% of the patients [5]. It prolongs the hospital stay, increases the cost of care, hinders effective rehabilitation, and worsens the disease progression leading to an increased mortality rate [2,7]. Clinical diagnosis of HAP in stroke patients can be a diagnostic challenge as frequent use of aspirin obscures the fever which is a cardinal feature of HAP [1,5]. Moreover, dyspnea might be present in many patients due to other comorbidities. Additionally, chest radiographs might not be helpful in the early detection of HAP [1]. All these factors can make the clinical diagnosis of HAP in stroke patients an exacting challenge for neurologists [8].

According to international multidisciplinary guidelines, HAP after ischemic stroke encompasses all the organisms involved in lower respiratory tract infections that complicate stroke after one week [9]. Traditionally, impaired mechanical ventilation, cough reflex, aspiration from dysphagia, immobility, and weakened respiratory muscles have been associated with HAP after stroke [10]. However, contemporary research also reports a casual association of brain injury with infection due to an imbalance between the brain and peripheral immune response [11,12]. Brain injury alters the peripheral immune response causing lymphopenia, dysfunction of monocyte, lymphocytes, decreased tiers of inflammatory cytokines, and atrophy of lymphoid organs [12]. These mechanisms decrease immunity and enhance the susceptibility to infections such as HAP. Consequently, the increasing prevalence of HAP among stroke patients results in increased hospital burden and economic instability especially in resource-deprived developing countries [2]. Recent studies have reported a myriad of gram-negative organisms such as *Pseudomonas aeruginosa* and *Klebsiella pneumoniae* as a cause of HAP in stroke [8,11]. Even though the guidelines are continually changing based on recent research, the controversy in the literature intrigued us to evaluate the clinical and microbiological parameters involved in complicating stroke with HAP.

## Materials and methods

This retrospective cross-sectional study was conducted in the Department of Neurology, Imran Idrees Teaching Hospital, Sialkot Pakistan from August 2019 to March 2021. The study involved a total of 232 ischemic stroke patients with a confirmed clinical and radiological diagnosis of HAP. Clinical assessment was based on length of hospital stay, respiratory system examination (lung percussion and auscultation), fever, and the presence of purulent tracheal secretions. The radiological evaluation was made on a chest X-ray. Pertinently, the tracheal secretions of the patients were sent for microbiological analysis (culture and antimicrobial sensitivity). This was done to identify the causative bacteria and evaluate the antimicrobial susceptibility patterns.

Patients with ischemic stroke diagnosed with community-acquired or viral pneumonia were excluded. Patients receiving corticosteroid therapy or broad-spectrum antibiotics for the past two weeks were also excluded. Additionally, specimen showing multiple growths on microbiological analysis was also subjected to exclusion. The exclusion criteria ensured that the prevalence of the causative organisms is precisely reported. Patients were studied for multiple parameters including demographic details, comorbidities, clinical findings, organisms isolated, and antimicrobial susceptibility patterns. All data were obtained from the patient files, reports, and computer records. Categorical variables including frequency of dysphagia, outcome, GCS score category, and comorbidities were expressed as frequencies and percentages while numerical variables such as age were expressed as means and standard deviation. Thereafter, the data were entered and analyzed using Statistical Package for Social Sciences (SPSS, IBM, Armonk, NY, USA) version 25.0.

## Results

In the present study analyzing data of 232 patients, the mean age was 58.79 ± 8.62 years, with a range between 47 and 81 years. The baseline demographic variables across gender, marital status, smoking status, and comorbidities are delineated in Table 1.

**Table 1:** Baseline demographic details of the study participants. HAP: Hospital-acquired pneumonia

Parameter		Frequency	Percentages
Gender	Male	110	47.4%
	Female	122	52.6%
Smoking status	Smoker	78	33.6%
	Non-smoker	154	66.4%
Marital status	Married	215	92.7%
	Unmarried	17	7.3%
Comorbid conditions	Diabetes	143	61.6%
	Hypertension	121	38.4%
	Chronic obstructive pulmonary disease	35	15.1%
	Asthma	11	4.7%
	Chronic kidney disease	47	20.3%
	Ischemic heart disease	69	29.7%
	Cystic Fibrosis	2	0.8%
Time from admission to the diagnosis of HAP (hours)	48-72	71	30.6%
	72-96	88	37.9%
	96-120	54	23.3%
	More than 120	19	8.2%

A total of 71 patients died due to disease complications. A breakdown of various clinical parameters and chest radiograph findings of our study population are elucidated in Table 2.

**Table 2:** A breakdown of clinical parameters of the study participants.

Parameter		Frequency	Percentages
Dysphagia	Yes	154	66.4%
	No	78	33.6%
Mechanical ventilation	Yes	167	72%
	No	65	28%
Pathological findings on chest radiograph	Atelectasis	44	18.9%
	Consolidation	39	16.8%
	Lobar involvement	49	21.1%
	Pleural effusion	53	22.9%
	Cardiomegaly	23	10.0%
	Pulmonary infiltrates	13	5.6%
	Pulmonary congestion	11	4.7%
Spontaneous pneumothorax	Yes	19	8.2%
	No	213	91.8%
Glasgow coma scale (GCS) score	Less than 8	58	25.0%
	8-13	65	28.0%
	13-15	109	47.0%
Outcome	Dead	71	30.6%
	Alive	161	69.4%

Most of the isolated microorganisms from the patients were predominantly gram-negative rods. The organisms isolated from the culture plates are shown in Table 3.

**Table 3:** A descriptive analysis of isolated microorganisms from tracheal secretions.

Microorganism isolated	Total frequency	Percentage
Staphylococcus aureus	41	17.7%
Klebsiella pneumoniae	67	28.9%
Pseudomonas aeruginosa	54	23.3%
Escherichia coli	57	24.5%
*Acinetobacter *species	8	3.4%
*Enterobacter *species	5	2.2%

The spectrum of antimicrobial susceptibility of these isolated organisms showed that most organisms were resistant to commonly used antimicrobial agents. The percentages of resistant isolates against various antimicrobials are elucidated in Table 4.

**Table 4:** Percentages of resistant isolates against various antimicrobial agents.

Antimicrobial drug	Isolated microorganisms					
	Staphylococcus aureus	Klebsiella pneumoniae	Pseudomonas aeruginosa	Escherichia coli	*Acinetobacter *species	*Enterobacter *species
Methicillin	100%	100%	100%	100%	100%	100%
Amoxicillin and clavulanic acid	87.3%	94.1%	100%	81.5%	100%	100%
Cephalexin	100%	100%	100%	100%	100%	100%
Cefixime	78.4%	81.5%	100%	94.1%	87.5%	60.0%
Ceftriaxone	32%	41.0%	77.2%	40.3%	37.5%	60.0%
Cefepime	0.0%	28.3%	38.8%	14.0%	12.5%	0.0%
Piperacillin and tazobactam	0.0%	13.4%	16.6%	15.8%	12.5%	20.0%
Vancomycin	4.0%	82.1%	85.2%	98.1%	100%	100%
Imipenem	0.7%	5.9%	3.2%	0.0%	12.5%	20.0%
Meropenem	0.7%	0.0%	1.0%	0.0%	12.5%	0.0%
Aztreonam	2.4%	0.0%	0.0%	0.0%	0.0%	0.0%
Linezolid	0.0%	94%	100%	100%	100%	100%
Azithromycin	34.1%	5.9%	31.5%	15.7%	12.5%	20.0%
Gentamicin	53.6%	32.8%	40.7%	24.5%	12.5%	20.0%
Ciprofloxacin	0.0%	10.44%	22.2%	19.3%	12.5%	20.0%
Levofloxacin	0.0%	13.4%	46.3%	21.0%	12.5%	20.0%

## Discussion

HAP is one of the most serious and fatal infections that complicates stroke with a mortality rate of 20% to 50% [6]. This is consistent with the finding of our study that reports a mortality rate of 30.6%. In our study, dysphagia was reported in 66.4% of the patients which shows that dysphagia might have a high association with HAP after ischemic stroke. Dysphagia causes impaired swallowing that leads to aspiration of oral microbiota and gastric contents in the lungs which predisposes them to pneumonia [6]. The casual association of dysphagia with post-stroke HAP has also been proven in another study that reports dysphagia in 53.8% of HAP patients with a relative risk of 4.74 times [13]. Similarly, mechanical ventilation is another clinical parameter found to be associated with HAP in stroke patients. In our study, a total of 72% of the patients with HAP were mechanically ventilated. The endotracheal tube acts as a conduit in the colonization of oral flora to the lung parenchyma thus, causing HAP [10]. In regards to pathological findings on chest radiography, atelectasis, consolidation, lobar involvement, pleural effusion, pulmonary infiltrates, and congestion was seen in less than 20% of patients. These findings were consistent with another study [14]. Chest radiographs are usually normal in the early stages of HAP, which might explain the low prevalence of these typical findings [1,14].

The microbiological analysis of the tracheal secretions showed that the most commonly isolated organisms causing HAP in stroke patients were *K. pneumoniae*, *P. aeruginosa*, and *Escherichia coli*. Many different studies also report similar findings with *P. aeruginosa* and *K. pneumoniae* as the most common organisms responsible for HAP in ischemic stroke [7,8,11]. Additionally, methicillin-resistant *Staphylococcus aureus*, *E. coli*, and *Acinetobacter baumannii* were also found to be involved in HAP [7,8,15].

Antibiotic-resistant organisms are increasing at an alarmingly high level across the globe since the past decade. Upon evaluation of antimicrobial resistance, we found that all organisms were completely resistant to methicillin and cephalexin. A similar finding has been stated in another study that reported high resistance to penicillins and first-generation cephalosporins [16]. On the contrary, our study shows that fewer percentage of isolates were resistant to fourth-generation cephalosporins, fluoroquinolones, and carbapenems. Furthermore, many multidrug-resistant organisms were found to be effectively treated by meropenem, imipenem, and aztreonam. Literature also proves the efficacy of these antibiotic regimens in the treatment of drug-resistant HAP [17]. Nonetheless, the drug-resistant profile of *P. aeruginosa* and *K. pneumoniae* in our study are extremely alarming as these organisms are highly prevalent. Another study reporting the frequency of extended drug-resistant organisms in a hospital setting emphasizes on rational use of antibiotics due to carbapenem-resistant gram-negative organisms [18].

Antibiotic resistance is rising at an alarmingly high level and the situation is worsening in the developing world. Excessive over-the-counter availability, irrational use, and unfettered supply chains contribute to gross misuse of antimicrobials especially in developing countries [18,19]. Moreover, increased self-medication is another contributing factor to this dilemma in developing countries [17,19]. In contrast, antibiotic therapy is targeted, rational and efficient in developed countries where the magnitude of the problem is somewhat minuscule [19].

For the prevention of antimicrobial resistance and the spread of resistant organisms, we have the following recommendations. Enhancing hygiene and sanitation can limit the spread of resistant organisms, thus, decreasing the use of antibiotics [20]. The emerging strategies of using alternative therapies like probiotics and maintenance of hygiene have also helped in preventing resistance [18]. The most important of all the preventive measures is the rational and specific use of antibiotics according to the laboratory reports and prescriptions [20,21]. A single-centered cross-sectional study design and convenience sampling technique are few limitations of our study. Nonetheless, the results of our study should be given serious considerations as treatment of resistant organisms causing HAP is a great medical challenge for neurologists. Further research on the topic in the form of systematic reviews and meta-analyses might help in the curation of specific guidelines for optimal treatment of such patients.

## Conclusions

Dysphagia and mechanical ventilation are important clinical parameters present in stroke patients with HAP. Gram-negative isolates including *K. pneumoniae*, *E. coli*, and *P. aeruginosa* are the most common causes of HAP in patients of ischemic stroke. Methicillin-resistant *S. aureus* was the most frequent gram-positive isolate from these patients. *Enterobacter* and *Acinetobacter* species were the least prevalent. The resistance of these organisms against commonly used antimicrobial agents is alarmingly high which necessitates the need for alternative therapies along with rational utilization of antibiotics. More studies on the topic will aid in the development of specific guidelines to combat this emerging problem.
